# In vitro spermatogenesis in two‐dimensionally spread mouse testis tissues

**DOI:** 10.1002/rmb2.12291

**Published:** 2019-08-13

**Authors:** Mitsuru Komeya, Hiroyuki Yamanaka, Hiroyuki Sanjo, Masahiro Yao, Hiroko Nakamura, Hiroshi Kimura, Teruo Fujii, Takuya Sato, Takehiko Ogawa

**Affiliations:** ^1^ Laboratory of Biopharmaceutical and Regenerative Sciences, Institute of Molecular Medicine and Life Science Yokohama City University Association of Medical Science Yokohama Japan; ^2^ Department of Urology Yokohama City University Graduate School of Medicine Yokohama Japan; ^3^ Department of Mechanical Engineering Tokai University Hiratsuka Japan; ^4^ Institute of Industrial Science University of Tokyo Tokyo Japan

**Keywords:** acrosin, germ cells, organ culture techniques, spermatogenesis, testis

## Abstract

**Purpose:**

Mouse in vitro spermatogenesis is possible with classical organ culture methods, by placing the testis tissue at the interphase between culture medium and air. In this condition, however, a tissue piece tends to round up to be compact, whose central region suffers from shortage of nutrients and oxygen. In this study, the authors improved the culture condition by spreading each tissue thin and flat, by which they were able to get better access to the oxygen and nutrients.

**Methods:**

Immature mouse testis tissues placed on agarose gel block were forced to spread flat by covering with a polydimethylsiloxane (PDMS) ceiling chip (PC chip). They were then cultured for weeks and evaluated by the transgene expression of *Acr‐Gfp*, which reflects the progression of spermatogenesis.

**Results:**

Testis tissues covered with PC chip initiated and maintained spermatogenesis in its wider region than those without PC chip covering. Flow cytometric analysis demonstrated that the PC method yielded more numerous meiotic germ cells than those without PC. Immunohistochemical examination confirmed the authentic histological figure of spermatogenesis from spermatogonia up to round or elongating spermatids.

**Conclusions:**

The PC chip method is simple and effective to improve the efficiency of in vitro spermatogenesis in the organ culture system.

## 1 INTRODUCTION

Using a classical organ culture method, according to gas‐liquid interphase principle, we succeeded in reproduce mouse spermatogenesis in vitro in 2011.^1^, ^2^ The method is quite simple in that immature testis tissue pieces were placed on the agarose gel block which was half‐soaked in the culture media. This agarose gel culture method (AG) was repeated by several researchers and became a reliable culture method for in vitro spermatogenesis.^3^, ^4^, ^5^, ^6^ However, the efficiency and duration of in vitro spermatogenesis were not comparable to those in vivo. To improve its efficiency, we adopted microfluidic technology to construct devices for culturing testis tissues. Our microfluidic device included channels for medium to flow and a chamber for tissue in which testis tissues were set in spread flat. As the medium and the tissue were separated by a porous membrane in large area of the flat tissue, molecular exchanges between them should have been efficient. Oxygen, on the other hand, permeated through the polydimethylsiloxane (PDMS), a popular material for the microfluidic device, and reached to the tissue. Thus, almost every part of the tissue in the device enjoyed nutrients and oxygen efficiently and evenly. In fact, the spermatogenesis was maintained in high efficiency for more than 6 months, along with testosterone production, which is another important function of the testis.^7^, ^8^ Based on this experience, we considered that flattening testis tissue per se could provide an advantageous microenvironment for tissues. Then, in our previous study, we molded PDMS into a small board‐shaped chip with a wide, shallow dent on one side and placed it dent‐side down over tissues set on agarose gel block. Neonatal mouse testis tissues cultured under this PC chip for a week showed significant volume expansion, which is a reflection of physiological testis growth.^9^ This was due to the supply of nutrients and oxygen evenly over a wide range, thus ridding the tissue of central necrosis and degeneration that generally happen in three‐dimensional cultures, including the AG method.

In the present study, we tested whether the PC method is also advantageous for in vitro spermatogenesis over the AG method. The master mold for PC chip production was made with materials all easily available, such as adhesive tapes. As expected, tissues under PC chips spread flat on the agarose gel and showed relatively even expression of marker GFP of spermatogenesis. Total number of meiotic germ cells per tissue was significantly higher in the PC group than in the AG group, owing to little central degeneration in PC group.

## 2 MATERIALS AND METHODS

### 2.1 PDMS ceiling chip

**Figure 1:**
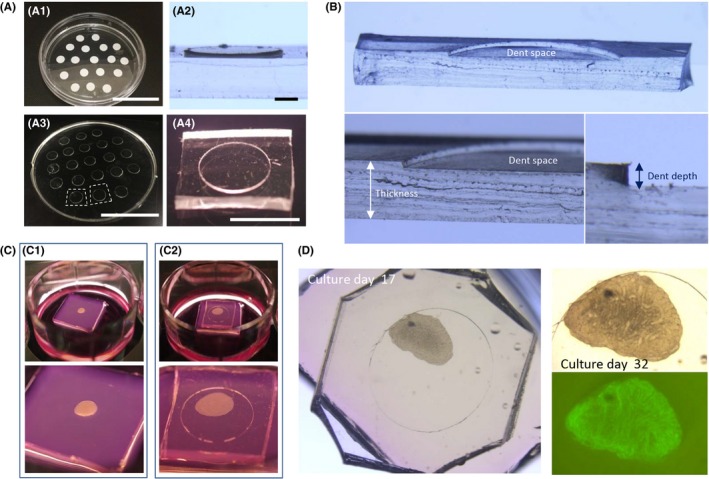
PDMS ceiling chip. A, The handmade master mold for PC chip. White vinyl tape cut out round was pasted on the bottom of 10‐cm culture dish, which served as master mold (A‐1). Looking closer, the tape produces a plateau whose height depends on the tape type and number of tapes piled (A‐2). PDMS prepolymer was poured in over the master mold and cured, followed by peeling off (A‐3) and cutting into pieces (A‐4). B, A PC chip cut in half vertically, showing its dent space, thickness, and dent depth. C, A mouse testis tissue before and after PC chip covering, left row and right row, respectively. D, A culture experiment with PC chip of 200 µm dent depth, on culture days 17 and 32. On day 32, the GFP expression was observed in the whole area of the tissue. Scale bar: 5 cm (A‐1, 3), 1 mm (A‐2), and 5 mm (A‐4)

**Figure 2:**
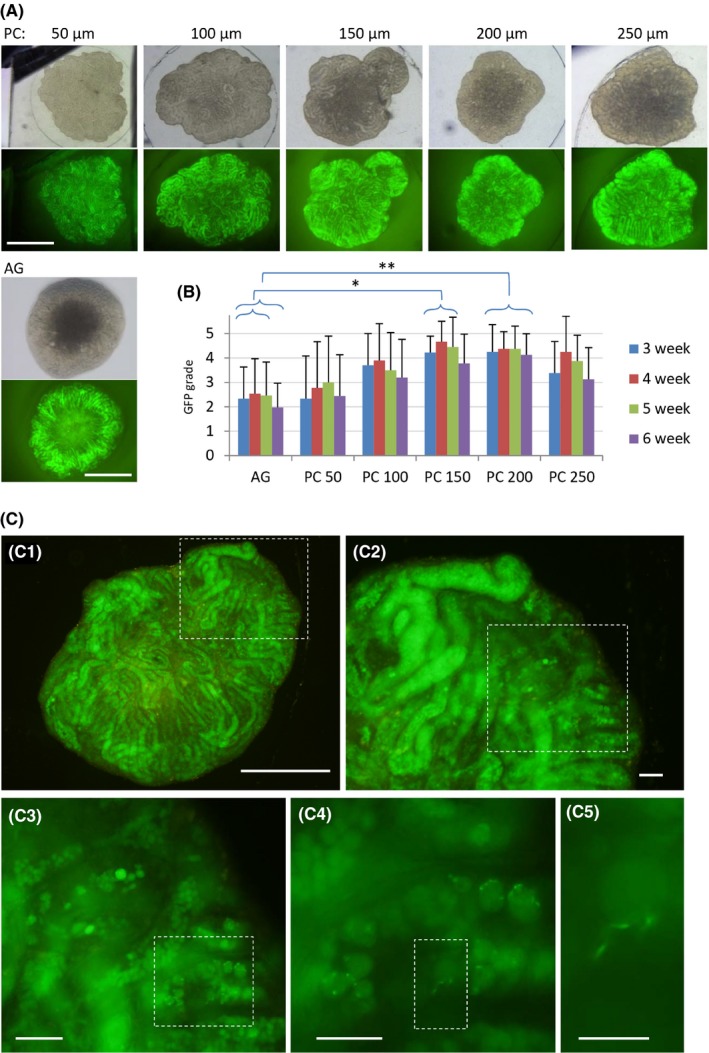
PC chip of different dent depths and in vitro spermatogenesis. A, The GFP expression of the cultured testis tissues with PC chips having different dent depths, 50, 100, 150, 200, and 250 µm, and without PC, namely the AG method. B, GFP grade was monitored for 7 weeks. PC groups of 150 and 200 µm dent depth each showed significantly higher GFP grade over the AG method in 3‐5 weeks and 3‐6 weeks, respectively. C, Testis tissues cultured under PC chip of 200 µm dent showed widely spread GFP expression with scattered signs of haploid germ cell formation at 6 weeks of culturing. The area of dashed rectangle is enlarged on the right or left lower panel sequentially. The right bottom picture represents GFP aggregated to the acrosome appearing as a cap‐like figure indicating round spermatid formation. Scale bars: 1 mm (A), 500 µm (C‐1), 100 µm (C‐2, C‐3), 50 µm (C‐4), and 20 µm (C‐5)

**Figure 3:**
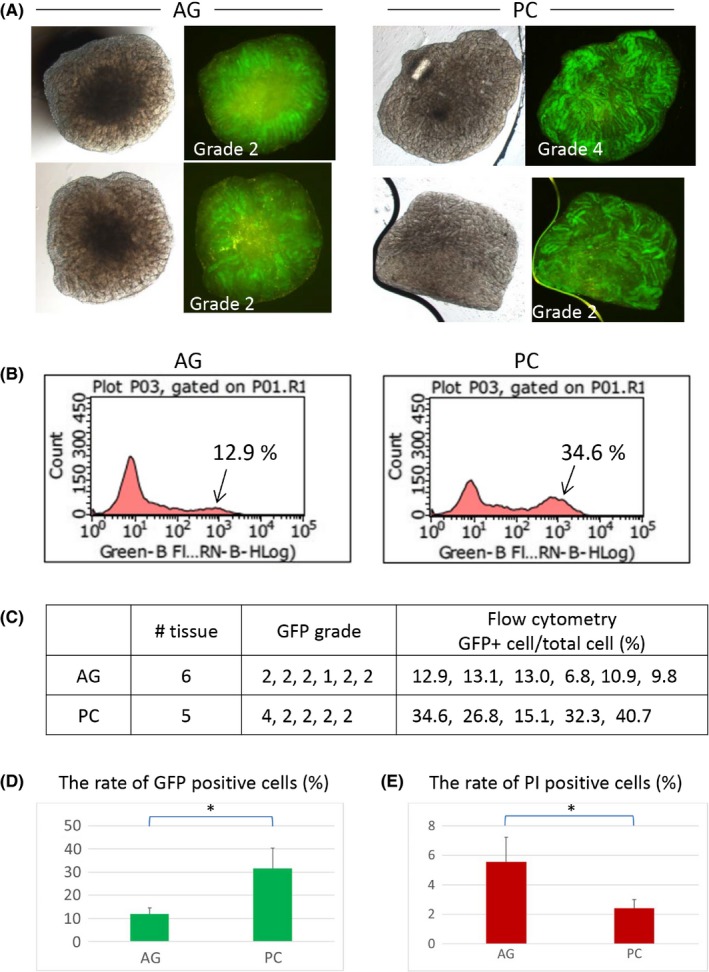
Efficiency of in vitro spermatogenesis with PC chip. A, Testis tissue of 4.5 dpp mice were cultured with the AG method, six tissues, or the PC method, five tissues, for 38 days. Two samples in each group are presented as typical examples. B, The cultured tissues were enzymatically dissociated and applied for flow cytometric analysis to count GFP‐positive cells among total viable cells. C, Summary table of data on six and five tissues, AG and PC, respectively. D, Average percentages of GFP (+) cells in the AG and PC groups. PC produced a significantly higher number of GFP (+) cells (*). E, Average percentages of PI (+), inviable, cells in the AG and PC groups. They were significantly different (*)

**Figure 4:**
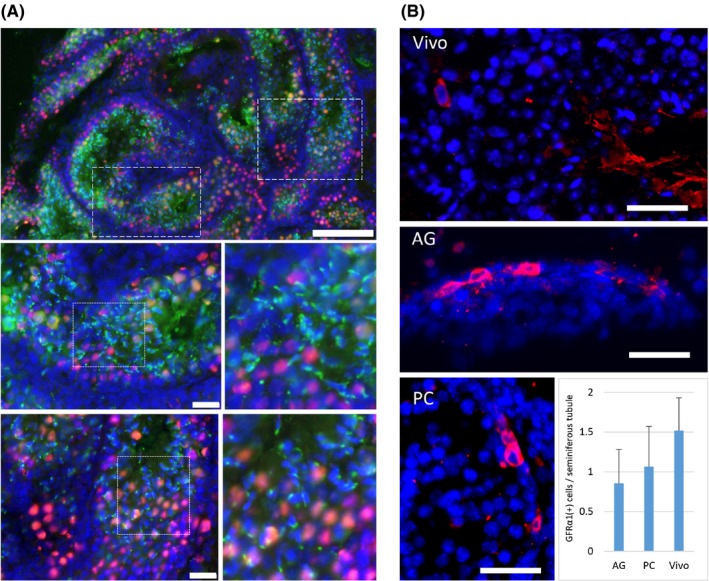
Immunohistochemical evaluation. A, Immunohistochemical examination showed that both GFP (green)‐ and TRA98 (red)‐positive cells were present in the seminiferous tubules of the cultured testes with PC chip. Areas of dashed rectangles in the left top panel are enlarged in the panels below. Areas of dotted rectangles are enlarged on the right each, showing many round and elongating spermatids. B, GFRα1‐positive cells, corresponding to the undifferentiated spermatogonia, including spermatogonial stem cells, were identified in the cultured testes with PC of 200 µm dent depth, AG, and testis tissue just taken out from an adult mouse (Vivo). Scale bar: 100 µm (A; top), 20 µm (A; middle and bottom), and 20 µm (B)

The PDMS ceiling (PC) chip was produced by mixing PDMS prepolymer and curing reagent (Silpot 184; Dow Corning) at a 10:1 weight ratio. The mixture was poured over the mold master, then placed in a vacuum chamber for degassing, and moved to an oven to be heated at 72°C for 1.5 hours for curing. As for mold master, conventional photolithography and soft lithography techniques are usually used. In this study, however, handmade master mold was adopted. We used two kinds of tapes, vinyl tape and cellophane tape, whose thickness is about 200 and 50 µm, respectively. These tapes cut out round, 5 mm in diameter, by a dermapunch were pasted on the bottom of 100‐mm culture dish (Figure 1A‐1). This dish served as a mold master, which can be used repeatedly. After cooling down, solidified PDMS was peeled off from the master (Figure 3A‐3). This PDMS disk was diced into individual chips using a cutter‐knife (Figure 4A‐4). The thickness of chips depends on the amount of PDMS poured in (Figure 1B). When we used 10 mL of PDMS prepolymer, the thickness became 1.8‐2.0 mm. The dent depth depends on the thickness and number of tape pasted on the 10‐cm dish (Figure 2A‐2, B). In order to make 100, 150, and 250 µm dent depth, round‐cut tapes were pasted on top of each other accordingly.

### 2.2 Animals

*Acr‐Gfp* transgenic mice,^10^ which were provided by RIKEN BRC through the National Bio‐Resource Project of MEXT, Japan, were used as a testis tissue source. Males homozygous for *Acr‐Gfp* were mated with females, which were either homozygous, heterozygous, or the wild type, to produce pups. The testis tissues of 0.5‐9.5 days postpartum (dpp) were used. Strain background was the mixture of C57BL/6 and ICR. The GFP expression in the testicular germ cells of *Acr*‐*Gfp* transgenic mice starts at mid‐pachytene stage. Then, at the early round spermatid stage, GFP in the cytoplasm accumulates to proacrosome and appears as a condensed aggregation adjacent to the nucleus. During spermatids transform from round to elongating, the shape of aggregated GFP also changes as the acrosome changes its structure. Thus, this GFP expression reflects the progression of spermatogenesis into and beyond meiotic stage.^10^, ^11^ Mice were housed in air‐conditioned rooms at 24 ± 1°C and 55 ± 5%, with a 14‐hour light/10‐hour dark lighting cycle. The mice were kept in a specific pathogen‐free room. Commercially made hard pellets (MF; Oriental Yeast) were fed ad libitum. Drinking water was acidified to pH 2.8‐3.0 using HCl. All animal experiments conformed to the Guide for the Care and Use of Laboratory Animals and were approved by the Institutional Committees of Laboratory Animal Experimentation (Animal Research Center of Yokohama City University, Yokohama, Japan).

### 2.3 Incubation of testis tissues

Testes were decapsulated and the tissue was divided into several pieces of appropriate size with forceps. Tissues were placed on the agarose gel block (1.5% w/v) half‐soaked in the culture medium in each well of a 12‐well plate (CELLSTAR® Tissue Culture Plates; Greiner Bio‐One).^1^, ^12^ They were incubated as they were, AG group, or covered with PC chip, PC group. The culture medium was adjusted up to around half of the height of the agarose gel (approximately 0.5 mL/well). All medium was changed once a week. The culture incubator was supplied with 5% CO_2_ in air and maintained at 34°C. The α‐modified Eagle medium (αMEM; Invitrogen: 12000‐022) supplemented with AlbuMAX (Invitrogen: 10828‐028), 40 mg/mL at final concentration, was used as culture medium.

### 2.4 Stereomicroscopic examination on GFP expression

Tissues in culture were observed once a week under a stereomicroscope (Leica M 205 FA; Leica). The proportion of the area showing GFP expression was recorded to evaluate the efficiency of spermatogenesis. It was classified into 6 grades, 0‐5, based on the expression area: 0, ~20, ~40, ~60, ~80, and‐100%, respectively. This GFP grading scale faithfully corresponded to spermatogenic progression, as confirmed in previous studies.^7^, ^12^ To identify haploid cells with an acrosome cap structure, cultured tissues were observed with an inverted microscope applying the GFP‐excitation light (Olympus IX 73; Olympus).

### 2.5 Immunohistochemical examination

Tissues fixed with 4% paraformaldehyde in PBS were cryoembedded in OCT compound (Sakura FineTechnical) and cut into 7‐µm‐thick sections. Nuclei were counterstained with Hoechst 33 342 dye. Specimens were observed with a microscope (Olympus IX 73; Olympus). The following were used as primary antibodies: chicken anti‐GFP antibody (1:1000; Nacalai Tesque, Inc), rat anti‐TRA98 antibody (1:200; Abcam), and anti‐GFRα1 antibody (1:200; R&D Systems). The secondary antibodies used were mouse anti‐chicken IgG and goat anti‐rat IgG, conjugated with Alexa 488 or Alexa 555 (1:200; Life Technologies).

### 2.6 Flow cytometry

The cultured tissues of *Acr‐Gfp* transgenic mice were digested by a two‐step enzymatic procedure to generate single‐cell suspensions.^13^ They were first incubated in phosphate‐buffered saline (PBS) containing 1 mg/mL collagenase at 37°C for 15 minutes. After the centrifugation (190 *g*, 5 minutes), the supernatant was removed. Then, 0.25% trypsin solution was added and incubated at 37°C for 15 minutes. After the centrifugation and removal of the supernatant, cell pellets were resuspended in PBS supplemented with 5% FBS and stained by a final concentration of 2 μg/mL PI for several minutes. Samples were analyzed by the Guava® easyCyte flow cytometry system (Merck KGaA).

### 2.7 Statistical analysis

Results are presented as mean ± SD. Statistical analysis was performed using one‐way ANOVA followed by Turkey's test or Student's *t* test. *P* < 0.05 was considered significant.

## 3 RESULTS

### 3.1 The PC chip method supports in vitro spermatogenesis

Testis tissue mounted on the agarose gel block takes a dome‐like form (Figure 1C‐1), which does not basically change during the culture period of weeks. When PC chip was placed over the tissues, they changed the form to be flatter and fell in the space of the dent (Figure 2C‐2). As for a pilot study, we made PC chip using master mold with the vinyl tape intending to make the dent depth to be about 200 µm. This PC chip was placed on a tissue of 3.5 dpp mouse testis for culturing (Figure 1D). The *Acr‐Gfp* expression was observed as an indicator of spermatogenic progression and lasted for 8 weeks as usually observed without PC chip covering. In addition, the central area, where it usually does not show GFP expression, also expressed GFP, albeit a bit weaker, which suggested the nutrients and oxygen having reached to that region.

### 3.2 Effect of different dent depths on the tissue

We then interested in whether the depth of dent affects the progression of spermatogenesis because it affected the testis growth significantly in our previous study.^9^ We made PC chips with different dent depths, 50, 100, 150, 200, and 250 µm. As for evaluation, GFP expression extent overall in each tissue was used. Specifically, the cultured tissues were recorded as photograph every week and the proportion of GFP‐expressing region in each tissue was estimated and classified into 6 grades: 0, ~20, ~40, ~60, ~80, and ~100%.^12^ Contrary to the control of AG, whose central area usually lacked GFP expression, samples in the PC groups showed GFP expression rather uniformly throughout the whole tissue in many cases, although the central area is yet weaker in GFP (Figure 2A). The GFP expression grade recorded demonstrated that *Acr‐Gfp* started to appear in the third week of culture and lasted for 4 weeks thereafter. The GFP grade was highest when covered with PC chip of 150 or 200 µm dent depth. Although there were no significant differences against other PC groups, they were significantly higher than the AG group (Figure 2B). When samples were taken out for a closer observation with inverted microscope, GFP that accumulated to become a dot or crescent form was identified sporadically, which indicated the formation of round or elongating spermatid (Figure 2C).

### 3.3 Efficient spermatogenesis under PC chip

The above data indicated that tissues under PC chip enjoyed nutrients and oxygen as efficiently as those not having PC chip covering, namely the AG method. In addition, the central portion of the tissue, which is usually supposed to suffer from shortage of both nutrients and oxygen in the AG method, appeared to have better access to them in the PC method. To confirm this advantage in the PC method quantitatively, numbers of GFP‐expressing meiotic germ cells were counted using the flow cytometry. Tissue fragments of 4.5 dpp mouse testis were incubated for 38 days either under PC chip of 200 µm dent depth or with the AG method (Figure 3A). Then, tissues were digested with enzymes for cell isolation and applied for flow cytometry (Figure 3B). Although the GFP grade hardly distinguished a difference in the extent of spermatogenesis occurrence in each tissue piece, the data of flow cytometry gave subjective quantitative data (Figure 3C). Among total cells counted as viable, GFP‐positive cells were 12.0% and 31.6% in the AG and PC groups, respectively, showing significant difference (Figure 3D). The ratios of dead cells, which were identified as propidium iodide (PI)‐positive cells, were 5.55% and 2.42% in the AG and PC groups, respectively, also showing significant difference (Figure 3E). These results supported the above results that the PC method improved the efficiency of in vitro spermatogenesis as a whole.

### 3.4 Immunohistochemical evaluation

In order to confirm the spermatogenesis in more detail, immunohistochemical study was performed. Testis tissues of 5.5 dpp mouse was cultured for 54 days under a 150 μm PC chip. Each tissue was sectioned horizontally to make its maximum cut area and stained with the antibodies to TRA98, a pan‐germ cell marker, and to GFP, a marker for meiotic germ cell. Production of haploid cells was confirmed by the presence of condensed GFP forming a dot or cap‐like structure assembling in the center of the seminiferous tubules (Figure 4A). The maintenance of immature germ cells was also evaluated using the antibody of GFRα1, which was expressed in undifferentiated spermatogonia. Mouse testes, 2.5 dpp and 9.5 dpp, were cultured for 4 weeks in PC chip with 200 µm dent depth and without, namely AG. Three and two tissues in AG and PC, respectively, were cut into 14 and 11 slices, respectively. They were stained for GFRα1, and positive cells in the seminiferous tubules were counted to be 0.85 and 1.07 cells/tubule in the AG and PC groups, respectively. These numbers were about two‐thirds that in 4‐week‐old mice (1.52 cells/tubule) (Figure 4B). This indicated that spermatogonial population including spermatogonial stem cells maintain themselves under the culture condition, with the PC method as well as the AG method.

## 4 DISCUSSION

The efficiency and quality of in vitro spermatogenesis were improved by the microfluidic devices as previously reported.^7^, ^8^, ^14^ These devices are supposed to provide a better microenvironmental condition for the cultured tissues by flowing the culture media, which facilitates nutrient supply and the removal of waste products. In addition, as the tissue pieces were set spread in those microfluidic devices, tissue thickness was adjusted to be about 160 µm in each device, by which molecular exchanges of nutrients and waste products were also enhanced to be more efficient. Oxygen, on the other hand, was supplied through the wall of device made of PDMS. Although PDMS is oxygen permeable, it would have worked to reduce the toxic effect of oxygen, compared to the AG method in which tissues were exposed directly to the air in the incubator. We envisioned that these possible advantages of microfluidic device could be applicable in a more simplified manner. In particular, spreading tissue on the agarose gel by the PC chip was an idea and found to be effective to allow growth of immature testis in vitro.^9^ Naturally, we wondered if the PC method allows in vitro spermatogenesis, too. Thus, in this study, we examined first whether testis tissues under PC chip can induce and maintain spermatogenesis, as well as without PC chip. According to the *Acr*‐*Gfp* expression extent in the present study, it was concluded that PC chip did not show negative effect on spermatogenic progression. The area showing *Acr‐Gfp* expression was virtually wider in the PC group than in the AG group, because tissues in the PC group showed GFP expression even in its central area in most cases, even though its expression was slightly weaker than that of peripheral area. This result was confirmed by counting GFP‐positive cells by flow cytometric analysis after digesting tissues for single‐cell isolation.

As for the dent depth, which dictates the tissue thickness, 150‐200 µm appeared to be better than the rest in inducing wider areas of spermatogenesis in a tissue. This might seem reasonable considering the natural thickness of the seminiferous tubules, whose diameter reaches up around 200 µm when full spermatogenesis is taking place in it.^15^, ^16^ However, in reality, each seminiferous tubule does not grow up to that diameter under our culture condition. Even in vivo, the diameter changes significantly according to the environment and the amount of germ cells in them.^15^, ^17^ Thus, 150 µm or even shallower dent depth allowed the expression of *Acr‐Gfp*, which indicates spermatogenic progression up to mid‐pachytene stage of meiosis. We tentatively concluded that using PC chip with 150 or 200 µm dent depth would be better than other depth and without PC, namely the AG method, in culturing mouse testis tissue for inducing spermatogenesis. As the microfluidic device showed an advantage in maintaining spermatogenesis for longer period than AG the method,^7^ it is also an important issue whether the PC method also has such benefit. Thus, further studies are certainly necessary to define the exact advantages of the PC method in comparison with the AG method and culturing in the microfluidic devices.

In our previous study, PC chip was produced with the master mold, which was made with the conventional photolithographic technique. To make this step simpler, we used the tape‐based soft lithographic method in the present study. Specifically, culture dish and vinyl or cellophane tapes were substituted for the SU‐8 photoresist to produce the mold master. It was reported that this simple method would be advantageous in its quickness, biocompatibility, reliability, simplicity, inexpensiveness, and popularization for biomedical researchers. For instance, a patterned localization of endothelial cells in a culture vessel was easily produced by the tape‐based soft lithography and used for a wound‐healing assay by observing a collective cellular migration.^18^ However, there were only a few such reports using this method for biomedical research to our knowledge. Our present data demonstrated that tape‐based soft lithography method is easy to apply and useful in producing PC chip. The PC method was shown to be effective in inducing mouse spermatogenesis in a cultured tissue, which could be applicable to tissues other than testis for their architectural and functional development and maintenance.

## CONFLICT OF INTEREST

The authors declare that there are no conflicts of interest.

## HUMAN RIGHTS

This article does not contain any studies with human patients.

## ANIMAL RIGHTS

All animal experiments conformed to the Guide for the Care and Use of Laboratory Animals and were approved by the Institutional Committees of Laboratory Animal Experimentation (Animal Research Center of Yokohama City University, Yokohama, Japan).
